# Potential Role of microRNAs in inducing Drug Resistance in Patients with Multiple Myeloma

**DOI:** 10.3390/cells10020448

**Published:** 2021-02-20

**Authors:** Alessandro Allegra, Roberta Ettari, Vanessa Innao, Alessandra Bitto

**Affiliations:** 1 Department of Human Pathology in Adulthood and Childhood, University of Messina, 98125 Messina, Italy; aallegra@unime.it (A.A.); vinnao@unime.it (V.I.); 2 Department of Chemical, Biological, Pharmaceutical and Environmental Chemistry, University of Messina, 98100 Messina, Italy; 3 Department of Clinical and Experimental Medicine, University of Messina, 98125 Messina, Italy; alessandra.bitto@unime.it

**Keywords:** micro-RNA, multiple myeloma, chemoresistance, bone marrow microenvironment, antagomir, miRNA mimics, apoptosis

## Abstract

The prognosis for newly diagnosed subjects with multiple myeloma (MM) has significantly progressed in recent years. However, most MM patients relapse and after several salvage therapies, the onset of multidrug resistance provokes the occurrence of a refractory disease. A continuous and bidirectional exchange of information takes place between the cells of the microenvironment and neoplastic cells to solicit the demands of cancer cells. Among the molecules serving as messengers, there are microRNAs (miRNA), a family of small noncoding RNAs that regulate gene expression. Numerous miRNAs are associated with drug resistance, also in MM, and the modulation of their expression or activity might be explored to reverse it. In this review we report the most recent studies concerning the relationship between miRNAs and chemoresistance to the most frequently used drugs, such as proteasome inhibitors, steroids, alkylating agents and immunomodulators. The experimental use of antagomirs or miRNA mimics have successfully been proven to counteract chemoresistance and display synergistic effects with antimyeloma drugs which could represent a fundamental moment to overcome resistance in MM treatment.

## 1. Introduction

### 1.1. General Considerations on miRNAs and Chemoresistance

The prognosis for newly diagnosed subjects with MM has significantly progressed in recent years since new drugs, such as proteasome inhibitors, immunomodulatory drugs, heat shock protein inhibitors, immune-checkpoints inhibitors, selective inhibitors of nuclear export and monoclonal antibodies, have been launched on the market [1,2,3,4,5,6,7]. Nevertheless, several MM subjects continue to relapse and after several salvage therapies, the development of multidrug resistance provokes the onset of a refractory disease [8].

Processes able to cause drug resistance in MM are not well known and several genetic or acquired elements appear to participate in its onset. In fact, besides the biobehavioral transformations of myeloma cells in response to drugs, numerous findings propose that the direct adhesive relationships between the MM cells and the cells of the adjacent bone marrow milieu cause the onset of pro-survival signs conducting to drug resistance. This sort of drug resistance, called “cell adhesion-mediated drug resistance” (CAM-DR), is considered one of the most relevant systems able to provoke the escape of MM cells from therapeutic actions [9,10]. Therefore, clarification of the molecular systems inherent in CAM-DR may facilitate identification of new therapeutic tactics to overcome this problem. 

Bone marrow stromal cells (BMSCs), osteoblasts, osteoclasts, macrophages, endothelial cells, bone marrow adipocytes, and fibroblasts create a composite structure with extra cellular matrix proteins and growth factors capable of conversing with MM cells. This correlation may clarify the mechanisms of drug resistance in MM, as MM cells are sheltered by these cells [11,12,13], and stroma-induced defense of MM cells may operate via soluble elements discharged from BMSCs [14].

However, recently, it has been clarified that the microenvironment−MM cell relationship is not merely constituted of paracrine signals of soluble elements. A continuous and bidirectional exchange of information takes place between cells of the microenvironment and neoplastic cells to solicit the demands of cancer cells. Among these messenger molecules there are microRNAs (miRNAs) [15], a family of small noncoding RNAs (18–25 nucleotides) that regulate gene expression through base complementarity between the seed region of the miRNA and the 3′-untranslated region of the target mRNA. Depending on the degree of complementarity, miRNA connections can induce mRNA translational degradation or repression [16].

Extracellular miRNAs may be of two different types, such as microvesicle-(MV) free and MV-entrapped [17]. The first type, simply combined to argonaute 2 (AGO2) proteins, is the most frequent form and presents resistance to nucleases [18]. However, several cells envelop and deliver specific miRNAs into MVs. Lipid membrane vesicles are delivered from both MM cells and the cells of the bone marrow microenvironment and distribute their RNA and protein cargos, wherewith they modify gene expression in the neighboring cells [19,20]. Several forms of extracellular MVs have been reported, such as exosomes, which derive from the multivesicular bodies, the smaller shedding vesicles, which originate from the fission of the plasma membrane and the apoptotic bodies dropped from cells after apoptosis. Unlike from AGO2-correlated miRNAs, MV-entrapped extracellular miRNAs are transported to different cells where they control gene expression [19,20]. 

In any case, miRNAs are essential controllers of the human genes and regulate innumerable cellular pathways to direct cell proliferation. The miRNA alteration acts on cancer onset, diffusion and drug resistance [21,22,23,24].

### 1.2. Possible Mechanisms of the Action of miRNAs in Multiple Myeloma Chemoresistance

miRNAs can operate as oncogenic or tumor suppressor miRNAs depending on their targets, tumor suppressors or oncogenes, respectively [25], and several findings sustain the action of both types in ruling the drug response of different tumors. It has also been demonstrated that some miRNAs may control the drug response of MM cells via regulation of the apoptotic or proliferative pathways such as p53 [26,27,28]. In fact, several reports explored miRNAs and chemical resistance in MM drug resistant experimental models and recent papers evidenced that the p53-related signaling pathways are regulated by miRNAs, thus proposing a potential role of miRNAs in the drug response of MM cells. 

It is well known that p53 is a transcription factor that can control the expression of a myriad of miRNAs. This suggests that p53 can stimulate tumor suppressor miRNAs or inhibit some oncomiRNAs. The miRNAs activated by p53 essentially affect the antiapoptotic genes, thus enhancing the tumor suppressor activity of p53, or they can control p53 itself in a positive feedback loop. Contrariwise, miRNAs inhibited by p53 may target pro-apoptotic genes conducting to a reduction of the tumor suppressor action of p53 [29,30].

Leotta et al. demonstrated that adhesion of wild type p53 MM cells to BMSCs intensely increased miRNA-125a-5p level, while decreased p53 expression. Moreover, it was demonstrated that when wild type p53 harboring sensitive MM cells adhered to BMSCs, the mRNA of TP53 was reduced, while p53-targeting miRNAs, miR-125a, miR-125b, and miR-25 were increased. However, some p53-controlled miRNAs such as miRNA-15a and miRNA-16 were reduced, supporting the role of p53/miRNA interaction in stroma-induced drug resistance in MM [31].

As far other miRNAs, miRNA-181a and miRNA-181b are increased in MM cells with respect to normal plasma cells, as well as in drug-resistant MM cells with respect to drug sensitive cells and control the p53 tumor suppressor. miRNA-181a and miRNA-181b block programmed cell death and increase cell proliferation in MM cells [31,32,33,34]. Remarkably, ectopic expression of miRNA-137 increased the sensibility of the MM cells to bortezomib (BTZ) through augmenting p53 expression and decreasing ataxia telangiectasia mutated/checkpoint kinase 2DNA repair pathway (ATM/Chk2). miRNA-27a, miRNA-631, miRNA-324-5p, miRNA-155, miRNA-497, miRNA-520g, and miRNA-520h are also reported to be implicated in the chemoresistance of MM cells. As a matter of fact, the expression of these miRNAs resensitize MM cells to chemotherapy [35,36,37,38,39,40]; finally miRNA-34a was also positively correlated with wild type p53 function with particular actions on MM cell survival [41].

Cyclin-dependent kinase 5 (CDK5), a member of the CDK family, is regulated by p35/p39. Ballabio et al. reported that the concentration of miRNA-27a was reduced in BTZ-resistant MM cells and the ectopic expression of miRNA-27a resensitized these cells to BTZ via reducing CDK5, which acts as an oncogene and is correlated with reduced survival in MM patients [42,43].

**Figure 1:**
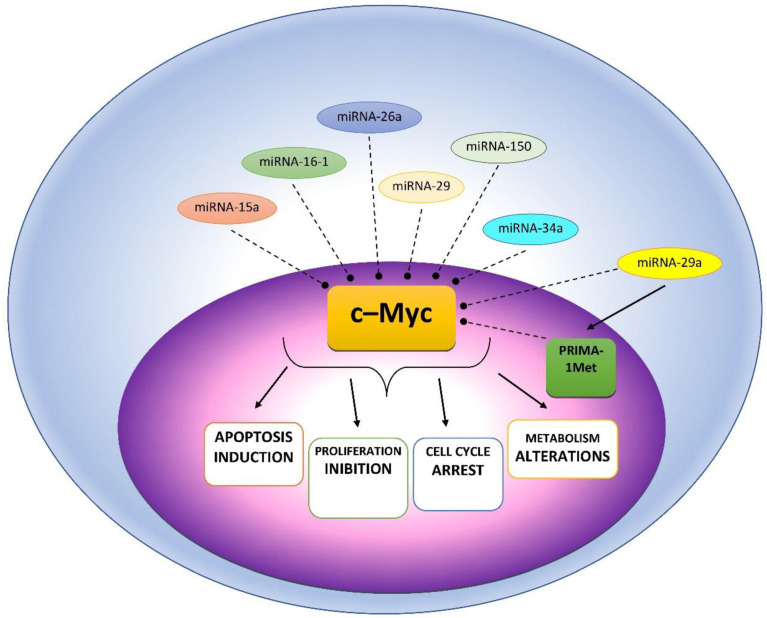
Possible mechanisms of c-Myc affecting miRNAs in MM cells.

Moreover, other miRNAs have been correlated with different oncogenic pathways essential for the onset of drug resistance, such as MYC, IFN and STAT [44]. For instance, it has been demonstrated that the proto-oncogene and transcription factor cellular myelocytomatosis oncogene (c-MYC) has an essential effect in MM onset [45] and was implicated in drug resistance [46] (Figure 1).

Blocking c-MYC stimulated programmed cell death in MM cells and the role of c-MYC-repressed miRNAs (miRNAs-15a/16-1, -26a, -29, -34a, and -150) in regulation of c-MYC-mediated activity on growth and cell death has been well described [47]. It is certain that increased expression of c-MYC has a fundamental role for chemoresistance. Interestingly, c-MYC causes the drug resistance of acute myeloid blast cells by blocking cell differentiation. Instead, in MM where plasma cells are terminally differentiated it acts via the interaction with different targets such as Myeloid cell leukemia-1 (MCL-1), an inhibitor of apoptosis, to cause drug resistance in different tumor diseases [48,49]. However, a recent report demonstrated that c-MYC regulated drug response in MM cells by acting on miRNA-29a, a tumor suppressor miRNA [50,51]. It was found that a p53-reactivating compound called PRIMA-1Met reduced c-MYC and MCL-1, but increased miRNA-29a in MM cells, and induced programmed cell death. c-MYC was recognized as a direct target of miRNA-29a, as an increase of miRNa-29a reduced c-MYC and blocked MM cell proliferation [52].

A mutation of other genes could also play a role in the onset of chemoresistance under the influence of miRNAs. Polycomb-like protein 3 (PCL3), also known as PHF19, is a polycomb-like (PCL) protein. PCL proteins are PRC2 (polycomb repressive complex 2)-related elements that develop subcomplexes useful for regulating PRC2 enzymatic activity, a histone methyltransferase activity that modifies histone H3 on lysine 27. Yu et al. demonstrated that increasing expression of the gene PHF19 caused MM cell proliferation and drug resistance in vitro and in vivo [53]. After chemotherapy with BTZ, epirubicin or melphalan, a reduction in MM cell death in PHF19 overexpression groups compared to the empty vector groups was observed. PHF19 was increased in drug-resistant primary cells from MM patients. Increasing PHF19 concentrations increased Bcl-xL and Mcl-1 presence in MM cells. miRNA-15a is able to target the 3′UTR of PHF19. It was demonstrated that the reduction of miRNA-15a caused an improved amount of PHF19 in MM cells. These data demonstrated that the miRNA-15a/PHF19 pathway has a central role in MM drug resistance [53].

Moreover, PHF19 stimulates the phosphorylation of EZH2 (enhancer of zeste homolog 2; the catalytic subunit of PRC2) via phosphoinositide-dependent kinase 1/protein kinase B (PDK1/AKT) signaling, and its increased expression was demonstrated also in MM [54]. EZH2 C-terminal SET domain comprehends the histone methyltransferase function that stimulates trimethylation of histone H3 lysine 27 (H3K27me3). In mature B-cells, EZH2 presence is reduced, causing an inhibition of EZH2 target genes. Contrariwise, an increased expression of EZH2 causes an augmented inhibition of EZH2 target genes and increased cell growth [54,55]. An elevated EZH2 amount in MM is linked to MM severity and bad prognosis [56,57,58,59]. It was reported that EZH2 is increased in drug-resistant MM cells [59] and a reduction of EZH2 provoked the contrary effect. Rastgoo et al. recognized miRNA-138 as a controller of EZH2. Experimental data demonstrated that RNA-binding protein with multiple splicing (RBPMS) is a target of EZH2. RBPMS silencing provokes resistance to MM cells and reestablishment of RBPMS by miRNA-138 resensitizes the resistant cells to drugs. Notably, in vivo release of miRNA-138 mimics n association with BTZ, causes relevant regression of MM in xenograft animal models [60].

**Figure 2:**
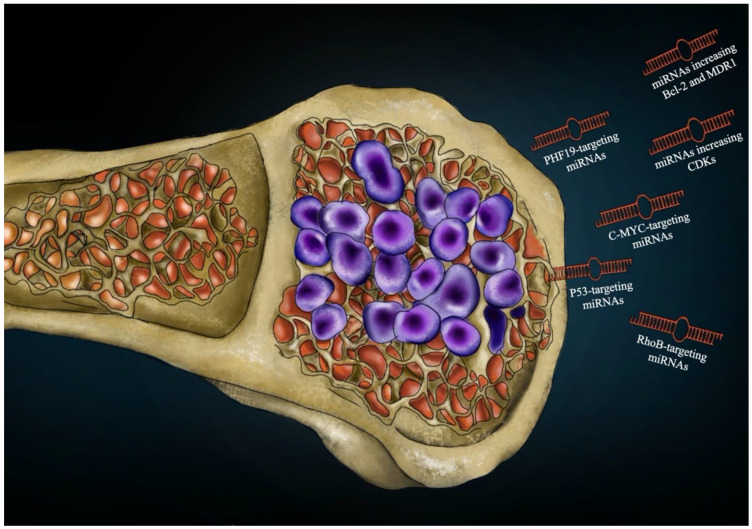
Possible targets of miRNA actions to induce chemoresistance in multiple myeloma.

The increased delivery of the antiapoptotic components of the B-cell lymphoma 2 (Bcl-2) family can provoke cell growth and chemoresistance [60]. Multidrug resistance is generally correlated with the increased expression of the MDR protein1 (MDR1), a membrane protein that belongs to the family of adenosine triphosphate (ATP)-binding cassette transporters and is recognized as the main factor in the transport of toxic compounds outside the cell [61,62]. It was demonstrated that miRNA-19a caused drug resistance by increasing Bcl-2 and MDR1 in MM cells in response to chemotherapy, and this process is controlled by the phosphatase and tensin homolog deleted on chromosome 10/phosphatidylinositol 3 kinase/Protein Kinase B (PTEN/AKT/pAKT)-signaling pathway [63]. miRNA-19a-3p might be employed as a potential marker to predict the efficacy of treatment in MM patients [64], (Figure 2).

In the following sections we report the most recent studies concerning the relationship between miRNAs and chemoresistance to the most frequently used drugs in patients with MM. Although most of the studies refer to proteasome inhibitors and in particular to BTZ, the onset of chemoresistance linked to the different expressions of miRNAs concerns all classes of drugs used, from steroids, to alkylating agents, to immunomodulators (Table 1).

### 1.3. miRNAs and Chemoresistance to Antimyeloma Drugs

Dexamethasone (Dex), a glucocorticoid (GC) is an essential drug employed in MM treatment and is generally part of the chemotherapeutic protocols for MM. However, high dosages of GC for a long time causes a GC receptor (GR) decrease, and a GC resistance. 

Zhao et al. utilizing Dex resistant (MM1R) and sensitive MM cell line (MM1S) demonstrated that the expression of miRNAs-221/222 was increased in GC-resistant cell lines and augmented MM cell survival through Bcl2 associated X/BCL2 antagonist/killer 1/BCL2 binding component 3. As a consequence, for overturning the drug-resistance to Dex, antagomir of miR-221/222 may be used and animals administered with Dex and miR-221/222 antagonist presented longer survival with respect to animals treated with Dex only [65].

Moreover, Dex-sensitive MM cell lines presented a greater amount of miRNA-15a with respect to MM1R resistant cells. Remarkably, the contact of MM cells with MM-BMSCs decreased miRNA-15a and -16 delivery in MM cells. Probably, the mechanism was cytokine-mediated as IL-6 produced by MM-BMSCs decreased generation of miRNA-15a and -16 in MM cells [66].

One study reported that miR-21 could also provoke drug resistance to Dex, doxorubicin, or BTZ in MM cells [40]. Moreover, authors demonstrated that adhesion to BMSCs increased miRNA-21 production in MM cells and caused drug resistance by targeting Ras homologous B (RhoB) gene. A recent study confirmed that the oncogenic miRNA-21 was a possible therapeutic target to overcome chemoresistance in MM [67].

Additionally, Murray et al. described an impressive antiapoptotic effect of miRNA-125b in the resistance to Dex action in MM cells [68]. Utilization of antisense miRNA-125b transcripts increased production of proapoptotic p53, reduced the delivery of antiapoptotic SIRT1 and increased Dex-induced apoptosis in MM cells [68]. Dex also caused an augmented expression of miRNA-34a, which is able to reduce SIRT1 deacetylase, thus causing inhibition of p53 [68].

Contrariwise, several microRNAs were reported to strengthen the apoptotic effect of Dex and this was exploited by Palagani et al. [93] who demonstrated a synergistic apoptotic effect of a combined treatment with low-dose GC and a synthetic vector of miRNA-150. Furthermore, miRNA-150 presented accessory effects acting on molecular chaperones, transcriptional factors, hormone receptors, and unfolded protein stress, reducing the probability of a GC resistance.

Recent studies demonstrated that miRNA-182 also has an essential action in GC resistance. Yang et al. reported that anomalous miRNA-182 production is associated with GC resistance in lymphoblastic leukemias though targeting the transcription factor forkhead class box O3a (FOXO3A) [69]. However, the action of miR-182 in MM is not well defined. In a study, it was reported that contact of H929 (another MM cell line) and MM.1S cells to fibronectin could increase miRNA-182 production and reduce programmed cell death 4 (PDCD4), which is essential for CAM-DR. Moreover, miRNA-182 was reported to negatively control PDCD4 production in H929 and MM.1S cells [70].

Conversely, the responsibility of miRNAs in the regulation of the efficacy of Immunomodulatory Drugs (IMiDs) such as Thalidomide, Lenalidomide and Pomalidomide has been inadequately studied. IMiDs demonstrated relevant antitumor effects in MM through reducing MM-cell proliferation in the bone marrow milieu and boosting immune effector cell activity. They are recognized to connect to the ubiquitin 3 ligase cereblon complex and so stimulating degradation of hematopoietic transcription factors IKZF1/3. Lately, AGO2, which has an essential effect in miRNA activity, was recognized as a cereblon binding partner and it was demonstrated that the steady-state concentrations of AGO2 are controlled by cereblon. In IMiD-sensitive MM cells treated with lenalidomide, apoptosis was observed together with an increase of cereblon and a reduction of AGO2 and miRNAs [71].

A significant influence of miRNAs was also found in the onset of chemoresistance phenomena to alkylating drugs. 

Munker et al. evaluated miRNA production in human myeloma cell lines, RPMI8226 and U266 and their resistant variants RPMI8226/Dox6 and RPMI8226/LR5, U266Dox and U266/LR7 [73]. 

It was reported that miRNA-221/222 inhibition may modify melphalan sensitivity in MM cells. Blocking of miRNAs-221/222 overwhelmed melphalan resistance and stimulated programmed cell death of MM cells in vitro. In fact, the therapy of severe combined immunodeficient/nonobese diabetic (SCID/NOD) animals carrying melphalan-refractory MM xenografts with locked nucleic acid (LNA) overwhelmed drug resistance [94].

A report demonstrated the justification of the combined administration of melphalan and locked nucleic acid-inhibitor (LNA-i)-miRNA-221 in drug-refractory MM subjects. The effect was due to the increase of PUMA, a (BH3)-only Bcl-2 member, and to a regulation of drug influx-efflux transporters, as SLC7A5/LAT1 and the ABC transporter ABCC1/MRP1. The powerful synergism was also substantiated in vivo by the employ of a systemically dispensed LNA-i-miR-221 [95]. BAX and BAK, two other p53-correlated genes, were also increased after inhibition of miRNAs-221/222 in vitro and in xenograft models. Authors proposed that as PUMA is a modulator of programmed cell death, it is possible that anti-sense-miRNAs-221/222 treatment should not only overwhelm Dex resistance but also resistance to other drugs employed in MM treatment, comprising lenalidomide. Remarkably, a report established that miRNA-221 production was controlled by RelB-p52 complex of the NFκB signaling pathway to which resistant MM cells are proposed to be addicted [74]. By reducing MM cells of RelB and p52, a relevant decrease of miRNA-221 was found. The study also demonstrated that RelB-p52 complex reduced expression of BMF, a proapoptotic gene which is a recognized target of miRNA-221. These findings identified the stimulation of anti-apoptotic pathways to be one possible mechanism of MM drug resistance induced by miRNAs-221/222.

However, most studies on the relationship between miRNA and chemoresistance in MM have been conducted on proteasome inhibitors and in particular on BTZ, a small-molecule proteasome inhibitor, normally employed to enhance the remission percentage and extend the overall survival of MM subjects. Although the initial response to BTZ is favorable, most subjects who primarily respond to BTZ acquire resistance to the drug. About 55−65% of subjects with relapsed MM are unresponsive to BTZ [96], and almost 20–30% of MM subjects have innate DR to BTZ [97,98,99].

Circulating exosome-correlated miRNAs were employed as prognostic markers to predict drug resistance, by evaluating responsive and BTZ-resistant MM subjects. In fact, a decrease of four exosomal miRNAs, comprising miRNA-15a-5p, -16-5p, -17-5p, and -20a-5p were reported in the BTZ-resistant MM subjects [75].

A diverse marker could be MiRNA-181a-5p, a target of long noncoding (Lnc) RNA MALAT1, whose interference reduced the production of miRNA-181a-5p and blocked the growth and adhesion of MM cells [79]. Several findings offer the suggestion that expression levels of miRNA-215-5p, -376c-3p and -181a-5p can be employed to predict the response to BTZ. 

MiRNA-15a/16-1, already mentioned above, was believed to possess tumor suppressor activities implicated in angiogenesis, cell differentiation, growth, or programmed cell death in numerous tumors including MM [100,101]. Preceding reports suggested that miRNAs-15a/16-1 reduction participated to the MM onset and played a role in drug resistance in myeloma cells. However, MM subjects with minor miRNA-15a expression were resistant to BTZ-based treatment, suggesting that miRNA-15a has a more relevant effect in drug resistance with respect to miRNA-16-1 in MM [65]. Li et al. demonstrated that subjects with a reduced amount of miRNA-15a had poor survival when treated with thalidomide or BTZ-based therapy, proposing that BTZ-based therapy did not enhance Progression Free Survival (PFS) and Overall Survival (OS) of subjects with miRNA-15a reduced expression [102,103].

In addition to the miRNAs mentioned above, others appear to have relevance in the determining of BTZ chemoresistance. In fact, in a different study, circulating exosomal miRNAs were extracted from newly diagnosed subjects with MM treated with BTZ and Dex, and autologous hematopoietic stem-cell transplant. Two miRNAs-derived exosomes, miR-18a and let-7b, were reported to be significantly correlated with poor prognosis in an independent mode even after correcting for the cytogenetic alterations [80]. Reduction of the delivery of exosomes by the sphingomyelinase inhibitor GW4869 augmented the sensitivity of MM cells to BTZ, causing an increased anti-MM response when BTZ and GW4869 were administered in a combined way [104].

As far the mechanisms by which miRNAs can cause resistance to BTZ, miRNAs can operate on oncogenes, such as specific protein 1 (SP1) or factors, such as B-cell activating factor (BAFF). For instance, tumor suppressors miRNA-29b and miRNA-27a- 5p regulate anti-MM action of BTZ in MM cells by aiming oncogenes such as SP1 [81]. Moreover, miRNA-202 regulates the BTZ effect via a decrease of the BAFF and JNK/SAPK pathway [105]. BAFF is a component of the tumor necrosis factors (TNF) superfamily and was recognized as an essential element affecting the proliferation of MM cells [106]. BMSCs increase BAFF in MM and it was demonstrated that levels of BAFF were augmented in BMSCs treated with miR-202 inhibitor. The production of Bcl-2 was decreased, and Bax was increased after miRNA-202 mimics transfection. An increased amount of miRNA-202 in BMSCs rendered MM cells more sensitive to BTZ. Moreover, miRNA-202 could reduce the stimulation of the NF-κB pathway in BMSCs. With respect to BTZ treatment alone, contemporary administration of BTZ and miRNA-202 mimics reduced MM cell survival and induced apoptosis [76].

In a different study, it was reported that levels of miRNA-21 were increased in MM cells after adhesion to BMSCs [40]. Treating U266 cells with miRNA-21 mimics made cells somewhat resistant to BTZ, Dex, and Dox. Authors demonstrated that NFκB may modulate miRNA-21 concentration. Blocking NFκB employing the inhibitor BAY had repressing actions on miRNA-21 expression. This suggested that miRNA-21 production in MM cells was controlled by NF-kB signaling, as also described for miRNA-21 in an experimentation on drug resistant B-cell lymphoma cells [77,78]. On the other hand, miRNAs such as miRNA-125a, b, -221/222, and -451 are increased in MM cells presenting drug resistance. The increased presence of these miRNAs diminished cell death caused by drugs such as BTZ and Dex; thus a reduction of these miRNAs could be a novel therapeutic approach to overwhelm drug resistance [76].

Abdi et al. reported that BMSCs reduced miRNA-101-3p expression and increased survivin (BIRC5) in MM cells [82]. Survivin was reduced by miRNA-101-3p overexpression and was reported to be a target of miRNA-101-3p. Increase of survivin augmented viability of MM cells when employed with antimyeloma drugs and blocking miRNA-101-3p through an anti-miRNA increased survivin. Moreover, the increase of miR-101-3p or the silencing of surviving stimulated programmed cell death in MM cells and sensitized them to antimyeloma treatment in the presence of BMSCs, overwhelming the stroma-induced drug resistance. In the same study, it was reported that HS-5 cells and MM BMSCs derived from MM patients provoked resistance to BTZ and carfilzomib (CFZ) in MM cells principally via direct cell−cell adhesion. Ectopic production of survivin made MM cells resistant to BTZ and inhibition of miRNA-101-3p enhanced survivin protein levels [82]. These results propose that BMSCs may utilize the miRNA-101-3p/survivin axis as a system to defend MM cells against antimyeloma treatment.

A diverse path capable of provoking BTZ resistance implicates the intervention of some integrins. CD47, an integrin-correlated receptor, is remarkably increased in MM drug resistance with respect to parental cells, and increased presence of CD47 is correlated with reduced progression free survival and overall survival. Rastgoo et al. demonstrated that miRNA-155 is present at small concentrations in drug-resistant MM cells and is a controller of CD47 [83]. An increase of miRNA-155 reduced CD47 expression in MM cells, causing the stimulation of programmed cell death of MM cells and an increased phagocytosis of MM cells. MiRNA-155 increased expression also re-sensitized drug-resistant MM cells to BTZ, causing cell death via targeting tumor necrosis factor-α-induced protein 8 (TNFAIP8), a negative controller of programmed cell death. Furthermore, it was demonstrated that the TNFAIP8 concentrations were greater in MM.1R cells with respect to MM.1S. Remarkably, ectopic expression of TNFAIP8 in the parental cell lines augmented resistance to BTZ by blocking caspase-8 activity and caspase-3. Moreover, increased expression of miRNA-155 in drug resistant MM cells decreased concentrations of TNFAIP8 [83].

The role of miRNA-155 in BTZ resistance was confirmed also by other studies. Amodio et al. evaluated miRNA-155 replacement as a possible anti-MM treatment in a xenograft model of MM. Moreover, in primary MM cells, they reported a negative correlation between miRNA-155 and the mRNA coding the proteasome subunit gene PSM_5, whose alteration has been involved in BTZ resistance [38].

The Snail family transcriptional repressor 1 gene (Snail1) was evaluated in MM cells from BTZ-resistant subjects. It was considerably correlated with the development of drug resistance systems. The mechanistic evaluations established that the increase of Snail1 production in BTZ-resistant MM cells directly augmented transcription of the intracellular multidrug resistance gene 1 (MDR1) and reduced P53 protein production via the Snail1/hsa-miRNA-22-3p/P53 pathway to decrease programmed cell death. By increasing MDR1 and reducing P53, Snail1 provoked the drug resistance of MM cells to BTZ, while Snail1 gene silencing efficiently enhanced drug sensitivity to BTZ treatment [84]. 

**Figure 3:**
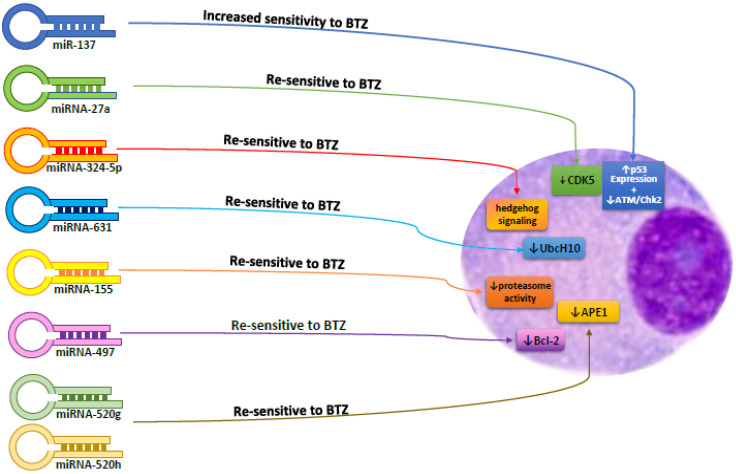
Effects of miRNAs on Bortezomib chemoresistance.

Other miRNAs capable of changing BTZ sensitivity are miRNAs 497 and miRNA 520. miRNA-497 is thought to be a tumor-suppressive miRNA in several tumors, being implicated in the control of proliferation and chemoresistance. An experimental study reported that miRNA-497 was reduced in MM subjects with respect to controls, and it was the one of four miRNA signatures recognized as having different concentrations that related with lenalidomide/dexamethasone therapy responses in a primary plasma cell leukemia clinical trial [85]. MiRNA-497 targets several genes able to influence apoptosis such as Bcl-2 [86,87,88,89,90], an increased concentration of miRNA-497 augmented MM cell apoptosis. Moreover, miRNA-497 enhanced the sensitivity of MM cells to BTZ. The combined administration of miRNA-497 and BTZ may augment drug sensitivity, providing a different therapeutic approach for MM [39] (Figure 3).

miRNA-520g and miRNA-520h belong to the miRNA-515 family [107], and several studies have evaluated their relationships with drug resistance. Ectopic production of miRNA-520g caused resistance to 5-fluorouracil- or oxaliplatin-provoked programmed cell death [107]. They were remarkably reduced in BTZ-resistant MM cells, and increased concentrations of miRNA-520g and miRNA-520h reduced production of recombination-related protein Rad51 and cell survival of BTZ-resistant MM cells by binding with Apurinic/apyrimidinic endonuclease 1 (APE1) mRNA. The combined increase of miRNA-520g and miRNA-520h blocked BTZ-resistant MM cell proliferation [40].

MiRNA-215-5p, miRNA-30e-5p and miRNA-29b-3p were also reported to have an essential action in the biology of MM and could have a role in chemoresistance [92].

However, more than the effect of a single miRNA capable of impacting a single pathway, chemoresistance appears to be the result of the action of a particular constellation of over- or down-expressed miRNAs [108]. In this sense, it is perhaps possible to define some specific profiles capable of predicting a condition of chemoresistance to BTZ. For instance, in a study, higher serum concentrations of miRNA-16-2-3p, miRNA-19b-3p, miRNA-29b-3p, miRNA-30e-5p, miRNA-122-5p, miRNA-143-3p, miRNA-148a-3p, and miRNA-215-5p, were described in BTZ-refractory subjects with respect to BTZ-sensitive subjects, while the serum concentrations of miRNA-30c-5p, miRNA-130a-3p, miRNA-151a-3p, miRNa-181a-5p, miRNA-191-5p, miRNa-328-3p, miRNA-376a-3p, miRNa-409-3p, miRNA-744-5p, miRNA-766-3p, and miRNA-1224-3p were inferior in refractory patients compared to the sensitive subjects [91].

## 2. Challenges and Future Perspectives

Progress of drug refractoriness is the most life-threatening condition in MM therapy, hindering most novel treatments. To overcome this problem, new experimentations are essential to investigate the mechanisms causing drug resistance in MM to obtain more effective therapeutic approaches.

The detection of miRNAs implicated in the regulation of the action of drugs is becoming a fascinating possibility, and the remarkable results that evidence that miRNAs are capable of changing the effectiveness of chemotherapeutics lead to a research area called “miRNA pharmacogenomics” [109]. 

However, there are several challenges in the identification of miRNAs as new biomarkers of chemoresistance. Various issues have to be taken into account, such as the selection of samples, the clinical conditions and the assay employed [110,111]. The origin of circulating miRNAs and their clinical significance should also be taken into account if we should use miRNAs as biomarkers [112,113,114]. A study by Pritchard et al. [115] emphasized that a huge amount of circulating miRNAs addressed as tumor markers by different studies, actually resembles those expressed by blood cells. 

From this point of view, the study of extracellular vesicle (EV)-microRNAs could be useful to explore the relationships between miRNAs and chemoresistance in MM patients. As reported above, exosomes are cell-derived vesicles, which transport nucleic acids, lipids, and proteins from their host cells [116]. Exosomes can be easily separated from biological fluids, such as blood and urine, and this offers a desirable new possibility to have a reliable biomarker for prognostic appliances [117]. Small EVs are believed to be a disease-specific source of circulating miRNAs since, in cancer patients, these have been associated to the sensitivity to different drugs [118].

A prognostic significance of EV-microRNAs has also been confirmed in several other hematological malignancies. Saito et al. observed a different EV-microRNA expression in subjects with myelodysplastic syndrome and acute myeloid leukemia during disease progression [119]. EVs might also have a prominent role in MM onset and progression by controlling endothelial cell functions, augmenting tumor cell growth, and increasing immunosuppression [120,121,122,123,124,125,126,127]. However, the most relevant effect seems to be exploited in cell–cell communication within the MM microenvironment [128]. Raimondo et al. reported a group of miRNAs expressed in EVs, from the MM cell line, and in the BM and plasma of subjects affected by smoldering myeloma and MM. They stated that miR-129-5p, which has an action on osteoblast differentiation, is enhanced in MM-EVs with respect to smoldering MM-EVs, thus proposing a specific enveloping associated with pathological grade [129], and in terms of clinical praxis, circulating EV-miRNAs from newly diagnosed MM subjects are a powerful marker for prognosis and outcome, such as let-7b and miR-18a. 

It is known that MM cells proliferate very fast and the hypoxic status in the BM may also be a cause of drug resistance, as proven by a study, during long-lasting hypoxia there was a massive release of EVs as compared to normoxic conditions [130]. 

Furthermore, it was reported that BM-Mesenchymal Stromal cell-derived EVs provoke drug resistance in MM cells [45]. Both naive and 5T33 BMSC-derived exosomes caused drug resistance to BTZ. BMSC-derived exosomes also modulated essential signaling pathways, including p38, p53, c-Jun N-terminal kinase, and Akt, and also caused drug resistance of human MM cells [131]. Finally, in a recent study, it was demonstrated a direct correlation between BTZ sensitivity and EVs-miR-1252-5p expressions which increased with sensitivity to BTZ [132].

However, our comprehension of the actions of noncoding material on chemoresistance processes is only just beginning and the possible interactions between different noncoding RNAs other than miRNAs such as long noncoding RNAs in the determining of chemoresistance are still to be discovered.

LncRNAs indicate a class of RNA transcripts larger than 200 nucleotides with no function of encoded proteins [133,134]. In the study of MM, lncRNAs were employed as competitive endogenous RNAs or a miRNA sponge. They contend with miRNAs to block their action, so modulating miRNA effects at the post-transcriptional level and controlling its target protein amount.

Few studies have stated the association between lncRNAs and drug resistance in MM. Reports demonstrated that MCL-1 had an effect on the proliferation of tumor cells evading drug activity. A study reported that lncRNA H19 was increased in the serum of MM subjects. It is known that miRNA-29b-3p is the downstream target gene, and MCL-1 is the downstream target protein of miR-29b-3p. So, it was hypothesized that MCL-1 may be implicated in the occurrence of drug resistance via H19. It was demonstrated that H19 decreased cell sensitivity to BTZ by operating as a miRNA sponge to reduce the expression of miRNA-29b-3p, increase MCL-1 transcriptional translation and block programmed cell death [135]. 

All these results may aid in obtaining new understanding of the molecular mechanisms of acquired BTZ resistance and generate novel drug targets for the therapy of MM, but numerous other problems remain to be solved. Some of the challenges in miRNA therapeutic schemes include miRNA stability, selective uptake by target cells via an active delivery system, potential off-targets and undesirable toxicities and the alteration of innate immune responses. Moreover, miRNA-based strategies must deal with the complexity of miRNA targetome, thus it could be useful to employ approaches based on “integrOmics” results, obtained from different technology platforms [136]. 

A further contribution could also be provided by nanotechnologies [137], that can surmount the biological obstacles for miRNA delivery and decisively take advantage of the huge potential of miRNA therapeutics. Nanotechnologies will permit extraordinary and selective penetration of miRNA-containing delivery vesicles into the BM microenvironment [138,139]. Moreover, the favorable pharmacokinetics of chemically modified miRNA inhibitors, such as LNA-oligonucleotides, offer exciting possibilities for clinical translation [140] to introduce miRNA therapeutics into the current therapy of MM. This could decrease the risk of drug resistance by affecting compensatory systems, drug distribution, metabolism, absorption, and excretion-related genes [141,142].

## 3. Conclusions

MM is a composite polygenic condition made by genetic alterations, modifications capable of influencing the onset, progression and response to therapy. 

Several reports demonstrated that miRNAs perform a fundamental action in BMSC-provoked drug resistance of MM cells. Since adhesion to BMSCs may regulate DNA-methyltransferase concentrations, this suggests new BMSC-driven epigenetic systems regulate miRNA expression in MM cells. Moreover, BMSCs were demonstrated to discharge miRNAs in exosomes, which could modify the phenotype of MM cells or other cells of the BM milieu by a paracrine manner [128].

Strengthening our comprehension of miRNA production and activity in the MM cell−BM relationship will help us to identify novel drug targets to overwhelm CAM-DR [143,144]. Several miRNAs are correlated with drug resistance, and the regulation of their production or action might be investigated to reverse it. The employ of antagomirs or miRNA mimics has been efficaciously demonstrated to have antitumor action and exhibit synergistic actions with antimyeloma drugs [64,66,73,145].

In any case, different approaches are currently under evaluation to operate on miRNA expression such as a decrease of upregulated oncomiRs and the replacement of downregulated tumor suppressor miRNAs. For instance, antisense miRNA inhibitors connect solely to the sense miRNA resulting in its inhibition. Moreover, locked nucleic acid linked to a phosphorothioate backbone is generally employed to increase the stability and the affinity of antagomirs to their target miRNAs. Other possibilities include miRNA sponges, which are transcripts that have numerous binding sites that avoid the binding of oncomiRs to mRNA. Another strategy could be represented by MASK, a synthesized oligonucleotide complementary to miRNA binding sites that prevents mRNA recognition [140,146,147,148,149].

Recently, to overcome chemoresistance to lenalidomide, a specific target was identified, Cereblon (CRBN), a brain-associated protein that interacts with DNA damage-binding protein-1, Cullin 4, and regulator of Cullins 1 to generate the functional E3 ubiquitin ligase complex that performs proteolysis via the ubiquitin-proteasome pathway. However, Lenalidomide can also have an action in MM cells by regulating miRNA levels and CRBN by binding to downstream protein AGO2 expression [72].

In conclusion, despite no clinical trials currently exploring the possibility of reducing chemoresistance through a modulation of miRNAs, this seems a fascinating and promising approach to improve and customize the therapy of MM. 

## Figures and Tables

**Table 1:** Effects of commonly used drugs on miRNA expression and final biological effect.

Drug-Resistance	Type of miRNAs	Action	Target Cells	Effect	Reference
Dexamethasone	miRNA-221	increased	MM1R	BAX/Bak1	[65]
	miRNA-222	increased	MM1R	BAX/Bak1	[65]
	miRNA-15a	decreased	MM1R	↑IL-6	[66]
	miRNA-16	decreased	MM1R	↑IL-6	[66]
	miRNA-21	increased	KMS-26, U-266, OPM-2, INA-6	RhoB	[67]
	miRNA-125b	increased	MM1S	Bak1/SIRT1 and↓p53	[68]
	miRNA-182	increased	H929, MM.1S	FOXO3A	[69,70]
**IMiDs**	AGO2	increased	IMiD-sensitive MM cells	cereblon	[71,72]
**Alkylants**	miRNA-221	Increased	RPMI8226/Dox6 and RPMI8226/LR5, U266Dox and U266/LR7	↑PUMA, SL7A5/LAT1, ABCC1/MRP1, BAX/Bak1, RelB-p52	[73,74]
	miRNA-222	Increased	RPMI8226/Dox6 and RPMI8226/LR5, U266Dox and U266/LR7	↑PUMA, SL7A5/LAT1, ABCC1/MRP1, BAX/Bak1	[73,74]
**Bortezomib**	miRNA-21	Increased		RhoB, NFkB	[67]
	miRNA-15a-5p	Decreased	BTZ-resistant cells	MAP-k, E2 enzymes	[75]
	miRNA-16-5p	Decreased	BTZ-resistant cells	MAP-k, E2 enzymes	[75]
	miRNA-17-5p	Decreased	BTZ-resistant cells	MAP-k, E2 enzymes	[75]
	miRNA-20a-5p	Decreased	BTZ-resistant cells	MAP-k, E2 enzymes	[75]
	miRNA-125b-5p	Decreased	BTZ-resistant cell	MAP-k, E2 enzymes	[76]
	miRNA-21-5p	Decreased	U266,	MAP-k, E2 enzymes	[40,77,78]
	miRNA-181a-5p	Increased	U266, MM.1S, RPMI8226	cell growth and MM cells adhesion	[79]
	miRNA-376c-3p	Increased	U266, MM.1S, RPMI8226	unknown	[79]
	miRNA-215-5p	Increased	U266, MM.1S, RPMI8226	unknown	[79]
	miRNA-18a	Increased	Primary MM cells	↓*HIF-1α*	[80]
	let-7b	Increased	Primary MM cells	↑*oncogenes CCND1, MYC, RAS*	[80]
	miRNA-29b	Decreased	Primary MM cells	↑oncogene *SP-1*	[81]
	miRNA-27a-5p	Decreased	Primary MM cells	↑oncogene *SP-1*	[81]
	miRNA-202	Decreased	U266	↑oncogene BAFF, JNK/SAPK, BAX	[76]
	miRNA-101-3p	Decreased	RPMI-8226, U266, MM.1S, OPM2, HS-5	↑survivin (BIRC5)	[82]
	miRNA-155	Decreased	Primary MM cells, MM1R	↑CD47, ↑TNF1IP8	[38,83]
	miRNA-22-3p	Increased	MM cells	↑Snail1/hsa, ↓*p53*	[84]
	miRNA-497	decreased	Primary plasma cell leukemia cells	Bcl-2	[85,86,87,88,89,90]
	miRNA-520g/h	decreased	BTZ-resistant MM cells	Rad51, APE1	[40]
	miRNA-16-2-3p	increased	Cells from BTZ-refractory subjects	multiple	[91]
	miRNA-19b-3p	increased	Cells from BTZ-refractory subjects	multiple	[91]
	miRNA-30e-5p	increased	Cells from BTZ-refractory subjects	multiple	[91,92]
	miRNA-122-5p	increased	Cells from BTZ-refractory subjects	multiple	[91]
	miRNA-143-3p	increased	Cells from BTZ-refractory subjects	multiple	[91]
	miRNA-148a-3p	increased	Cells from BTZ-refractory subjects	multiple	[91]
	miRNA-215-5p	increased	Cells from BTZ-refractory subjects	multiple	[91,92]
	miRNA-30c-5p	decreased	Cells from BTZ-refractory subjects	multiple	[91]
	miRNA-130a-3p	decreased	Cells from BTZ-refractory subjects	multiple	[91]
	miRNA-151a-3p	decreased	Cells from BTZ-refractory subjects	multiple	[91]
	miRNa-181a-5p	decreased	Cells from BTZ-refractory subjects	multiple	[91]
	miRNA-191-5p	decreased	Cells from BTZ-refractory subjects	multiple	[91]
	miRNa-328-3p	decreased	Cells from BTZ-refractory subjects	multiple	[91]
	miRNA-376a-3p	decreased	Cells from BTZ-refractory subjects	multiple	[91]
	miRNa-409-3p	decreased	Cells from BTZ-refractory subjects	Multiple	[91]
	miRNA-744-5p	decreased	Cells from BTZ-refractory subjects	multiple	[91]
	miRNA-1224-3p	decreased	Cells from BTZ-refractory subjects	multiple	[91]
**Carfilzomib**	miRNA-101-3p	decreased	RPMI-8226, U266, MM.1S, OPM2, HS-5	↑survivin BIRC5	[82]
**Doxorubicin**	miRNA-21	increased	BTZ-resistant MM cells	RhoB	[40]

## Data Availability

Not applicable.
